# Evaluation of Skeletal Muscle Function and Effects of Early Rehabilitation during Acute Heart Failure: Rationale and Study Design

**DOI:** 10.1155/2018/6982897

**Published:** 2018-03-12

**Authors:** Kinga Węgrzynowska-Teodorczyk, Agnieszka Siennicka, Krystian Josiak, Robert Zymliński, Monika Kasztura, Waldemar Banasiak, Piotr Ponikowski, Marek Woźniewski

**Affiliations:** ^1^Faculty of Physiotherapy, University of Physical Education in Wrocław, Wrocław, Poland; ^2^Center for Heart Disease, 4th Military Hospital of Wrocław, Wrocław, Poland; ^3^Department of Physiology, Wrocław Medical University, Wrocław, Poland; ^4^Department of Heart Diseases, Wrocław Medical University, Wrocław, Poland

## Abstract

**Background:**

Acute heart failure (AHF) is associated with disturbances of the peripheral perfusion leading to the dysfunction of many organs. Consequently, an episode of AHF constitutes a “multiple organ failure” which may also affect the skeletal muscles. However, the abnormalities within skeletal muscles during AHF have not been investigated so far. The aim of this project is to comprehensively evaluate skeletal muscles (at a functional and tissue level) during AHF.

**Methods:**

The study will include ≥63 consecutive AHF patients who will be randomized into 2 groups: ≥42 with cardiac rehabilitation group versus ≥21 with standard pharmacotherapy alone. The following tests will be conducted on the first and last day of hospitalization, at rest and after exercise, and 30 days following the discharge:* clinical evaluation*, medical interview, routine physical examination, echocardiography, and laboratory tests (including the assessment of NT-proBNP, inflammatory markers, and parameters reflecting the status of the kidneys and the liver);* hemodynamic evaluation*, noninvasive determination of cardiac output and systemic vascular resistance using the impedance cardiography;* evaluation of biomarkers reflecting myocyte damage*, immunochemical measurements of tissue-specific enzymatic isoforms;* evaluation of skeletal muscle function*, using surface electromyography (sEMG) (maximum tonus of the muscles will be determined along with the level of muscular fatigability);* evaluation of muscle tissue perfusion*, assessed on the basis of the oxygenation level, with noninvasive direct continuous recording of perfusion in peripheral tissues by local tissue oximetry, measured by near-infrared spectroscopy (NIRS).

**Results and Conclusions:**

Our findings will demonstrate that the muscle tissue is another area of the body which should be taken into consideration in the course of treatment of AHF, requiring a development of targeted therapeutic strategies, such as a properly conducted rehabilitation.

## 1. Introduction

A rapid increase in the incidence and prevalence of chronic heart failure (HF) has been observed in the 21st century. Despite the availability of efficient methods of HF management and improved prognosis, the quality of life of patients with this condition is still poor. This results from the necessity of frequent hospitalizations due to an exacerbation or natural progression of this condition. The progressive deterioration of the psychophysical condition of patients with HF constitutes not only a clinical but also a predominantly social problem [1–3].

It is both the sudden and the gradual exacerbation of the clinical signs that lead to acute heart failure (AHF), which is an essential component of the natural history of HF.

AHF affects about 2% of the European population and is still related to high mortality and high rehospitalization rates [1]. Multiple organ damage due to congestion and/or peripheral hypoperfusion is a common condition in the prevalence of AHF and is associated with increased mortality [4, 5]. Despite progress in modern medicine and the availability of novel diagnostic methods, the pathophysiology of AHF is still not completely understood. This results from the complexity of its etiology, heterogeneity of clinical presentation, and complex interactions between hemodynamic and neurohormonal disorders [1, 2, 4, 5]. Although there is a growing body of data on acute renal or myocardial injury [4–8], still very little is known about the AHF-related damage to other organs and tissues (e.g., the skeletal muscles).

The role of skeletal muscle abnormalities was frequently emphasized in the context of the complex pathophysiology of chronic HF. According to the “muscle hypothesis,” the predominant and clinically important sign of HF, that is, intolerance to physical exercise, is associated with functional and structural disorders of the skeletal muscles to a large extent [9, 10]. A reduction of muscle mass and muscular strength is a common phenomenon in patients with chronic HF [11, 12]. This is consistent with changes observed at a cellular level, that is, lower number and volume of mitochondria and mitochondrial crests, reduced level of glycogen, lower number and activity of oxidative and glycolytic enzymes, Krebs cycle disorders, enhanced degradation of phosphocreatine, and decreased pH. As a result of the phenomena mentioned above, the metabolism of patients with HF is mostly anaerobic, which leads to the earlier acidification of the muscles leading to their easier fatigability [13–16]. The increased activity of muscular ergoreceptors stimulates excessive ventilation, activation of the sympathetic system, and an increase in systemic vascular resistance [12, 17].

As part of this project, we are going to comprehensively examine whether AHF is associated with the injury of myocytes and with the significant functional impairment of skeletal muscles.

The normal work of skeletal muscles, that is, maintenance of muscle proper strength and endurance, is vital for the performance of activities of independent daily living. Moreover, any functional disorder, for example, of the respiratory muscles, can interfere with therapeutic processes. Finally, increased muscular fatigability limits independent functioning and leads to disability [18, 19].

AHF is a highly complex clinical syndrome. Hemodynamic disorders lead to the impaired perfusion of the heart, kidneys, liver, and central nervous system. These phenomena are consistent with a concept of “end-stage organ damage” according to which AHF constitutes a form of multiple organ failure [4, 20]. Noticeably, the role of skeletal muscle dysfunction has not been analyzed in this context to date.

Skeletal muscles constitute a considerable percentage of the total mass of the human body; therefore, their injury can manifest in altered levels of specific cellular injury markers or tissue hypoxia. A comprehensive analysis of the AHF-induced changes taking place at functional, tissue, and molecular levels will be important for clinical practice, diagnosis, and the targeted treatment of peripheral dysfunctions associated with AHF.

Assuming that AHF is associated with impaired perfusion and hypoxia of the muscle tissue, we postulate that AHF can lead to injury or necrosis of the myocytes and dysfunction of skeletal muscles as a result. Consequently, apart from the standard clinical evaluation of AHF, we are going to examine the processes taking place in skeletal muscles during the acute phase of the condition and 30 days following the stabilization of clinical status.

The function of skeletal muscles in patients who underwent cardiac rehabilitation during the acute phase has not been examined so far. This is especially challenging due to the highly complex clinical status of AHF patients. Knowledge of the function of skeletal muscles and their potential abnormalities is vitally important for physiotherapists, who work with clinically demanding AHF patients, being exposed to extremely high risks of clinical deterioration (including the risk of the death of the patient).

The proper use of various methods and forms of physical exercises can minimize the negative consequences of exacerbation, namely, limited mobility and decreased physical capacity, as well as decreased physical fitness. This may allow restoration of the functional capacity of patients [21–25].

This project will analyze the effects of rehabilitation on widely understood hemodynamic parameters.

Physical training constitutes part of the multidisciplinary management of HF [26–28]. Regular aerobic exercise is recommended for stable HF patients to improve their functional capacity and reduce the risks of HF-related hospitalization, in accordance with class A recommendations of the European Society of Cardiology (ESC) [1].

Experts point out the need for early patient activation after the episode of AHF decompensation, which may prevent disability [26]. The current researches concerning physical rehabilitation during hospitalization and early rehabilitation following discharge are not well represented in the literature.

Therefore, the role of cardiac rehabilitation during episodes of acute decompensated HF has been identified as a “critical evidence gap” and additional research on developing specific cardiac rehabilitation programs should be a priority [29].

Thus, the aim of this project is to comprehensively evaluate the skeletal muscles (at a functional and tissue level) and to evaluate the effects and safety of early rehabilitation programs in acute decompensated HF.

We developed this project in order to verify the following hypotheses:

1. Impaired perfusion of the muscles is reflected by their injury and significant functional abnormalities.
2. Early implementation of motor rehabilitation improves the perfusion and function of skeletal muscles.

*Research Questions*

1. Is AHF associated with impaired perfusion of skeletal muscles?
2. Is AHF associated with injury and dysfunction of skeletal muscles?
3. Is the proposed exercise protocol safe and does it exert a positive effect on the oxygenation and functioning of skeletal muscles?
4. How does the rehabilitation influence hemodynamic parameters such as cardiac output, stroke volume, arterial pressure, and systemic vascular resistance?

## 2. Study Sample

The study will include patients with AHF (defined according to the ESC criteria [1]) hospitalized at the Center for Heart Disease, 4th Military Hospital of Wrocław, and diagnosed with chronic systolic heart failure at least 6 months prior to admission.

Exclusion criteria comprise acute coronary syndrome, bacterial infection confirmed on the basis of clinical and laboratory criteria, preexisting chronic respiratory failure, necessity of mechanical ventilation, significant arrhythmia and conductivity disorders, anemia (hemoglobin < 9 g%), active neoplastic process, liver injury (AST, ALT > 3x reference level), and chronic kidney failure with creatinine clearance < 30 ml/min.

Patients whose functional or cognitive status or age will not allow performing the movement tasks will be excluded from the research project (e.g., hemiplegia, Alzheimer's disease, or depression).

Patients enrolled in the study will be randomized in a 2 : 1 ratio to the rehabilitation and control groups. We plan to enrol ≥63 patients, including ≥42 subjects in the rehabilitation group and ≥21 in the control group.

All study participants will provide informed written consent.

This study has been registered with Clinicaltrials.gov.

The research will be carried out as part of the Sonata 6 UMO-2013/11/D/NZ7/00922 research grant awarded by the National Science Centre.

## 3. Methods

The research will be performed at three time points: at admission, at discharge, and 30 days after the discharge. The study protocol is presented in Table 1.

1. Clinical evaluation: interview related to the whole medical history, physical examination including transthoracic echocardiography with an evaluation of the left ventricular ejection fraction, and laboratory indices of neurohormonal activation (NT-proBNP), inflammation (ESR, hsCRP), laboratory parameters reflecting the presence of selected HF comorbidities (urea, creatinine, uric acid, GGPT, AST, ALT bilirubin, proteins, albumins, TSH, fT4, and fT3,), lipid profile, and laboratory markers of hematological status (RBC, WBC, platelets, hemoglobin, hematocrit, MCV, MCH, MCHC, and RDW) [30].
2. Hemodynamic evaluation: transthoracic impedance cardiography (ICG), which is a safe, noninvasive diagnostic technique. By detecting and measuring the change of thoracic impedance over time, ICG measures hemodynamic parameters that could be applied for the diagnostics and monitoring of HF. The principle of the transthoracic bioimpedance use: the direct measurement of the baseline impedance, the velocity index, the acceleration index, the ventricular ejection time and the heart rate, and the early diastolic filling ratio. These parameters are used to compute the systolic volume, the cardiac output, and several other hemodynamic parameters. The system can be used at rest or while exercising [31–34].
3. Evaluation of muscular function by means of surface electromyography (sEMG); this is a noninvasive method, which uses electrodes placed on the skin alongside the running of muscle fibers to register electric processes (myopotentials) taking place in a working muscle. EMG signaling can be used to analyze the characteristics of muscle contraction, muscular coordination, recruitment, and frequency of motor unit activation. By analyzing the EMG parameters, that is, the frequency and amplitude of functional potentials, one can verify whether a given muscle is active and how its activation changes over time and can determine the maximum tonus of the muscle and its fatigability. Continuous monitoring of local muscle fatigue during performance is possible by measuring the myoelectric activity of muscles during isometric contraction as well as during dynamic work. One of the most often analyzed parameters of sEMG is the change of the mean and median frequency of the signal power spectrum and the change of amplitude (RMS, root mean square). Contraction-related muscular fatigability manifests as the shift in the frequency of the entire spectrum of power toward lower values, with a simultaneous increase in the amplitude [35–37]. In this project, we will analyze the fatigability of the vastus lateralis (VL), the vastus medialis (VM), and the rectus femoris (RF) muscles during isometric and dynamic work. Moreover, functional fitness will be assessed. The following tests will be conducted at discharge from the hospital and 30 days thereafter: 2-minute step test, determining the number of steps performed within two minutes; 30-second “chair stand” test, examining muscular endurance of the lower body (pertaining to the patient repeatedly getting up from a chair over a period of 30 seconds); “up and go,” determining power, speed, agility, and dynamic balance (involves getting out of a chair, walking 2,44 m around a pole, and returning); and handgrip test, examining isometric strength [38, 39].
4. Evaluation of muscle tissue perfusion (examined indirectly on the basis of oxygenation level): local tissue oximetry, also referred to as near-infrared spectroscopy (NIRS), will be used. This is a noninvasive method of direct continuous recording of perfusion in peripheral tissues. NIRS is based on the absorption of near-infrared photons by the so-called chromophores (e.g., hemoglobin). The degree of hemoglobin oxygenation is reflected by the amount of light absorbed by this molecule. Differences in the intensity of NIR waves absorbed by oxygenated and reduced hemoglobin are analyzed using algorithms based on the Lambert-Beer law. The measurements are taken by photosensors which can be easily placed on the skin. The NIR light emitted by the sensor's diode penetrates locally into the tissues underneath, and the level of hemoglobin in the microcirculation is determined by two detectors [40–42].

The evaluation of muscle tissue perfusion and muscle function will be assessed during 2 exercise/motor tasks: isometric and dynamic.

Before each motor task, 5-minute recording of the resting parameters will be carried out, followed by a 15-minute measurement after the task, during a recovery period.

The patient is informed that the exerted effort should be from a slight to a moderate level (<3) on Borg's Perceived Exertion Scale (0–10). All tests/exercises are conducted in the patient's hospital bed in their room.

Isometric exercise is performed in a semiseated position. This position is the most comfortable for patients with AHF.

The patient will perform two repetitions of maximal isometric contractions of the quadriceps thigh muscle, maximal voluntary contractions (MVC); then, after 2 minutes of rest, the patient will begin the exercise consisting of 1-2 seconds of contractions of the quadriceps thigh muscle with 50% MVC, followed by relaxation lasting two seconds. The subject will perform 45 repetitions of isometric contractions, followed by the MVC again.

Dynamic exercise is performed in a semiseated position. This position is the most comfortable for patients with AHF.

The examined lower limb will be bent at the knee joint at an angle of 45%, with the thigh resting against a wedge and heel resting on the bed. The patient's task is to extend the limb in the knee joint (raising the heel upwards). The patient will practice this for 90 seconds at a pace of 1 s : 1 s (extension : contraction, i.e., tensing/relaxing).

In addition to continuous and ongoing monitoring and recording of stroke volume (SV), heart rate (HR), cardiac output (CO), systematic vascular resistance (SVR), saturation, respiration rate, and muscular tissue oxygenation, the blood pressure value and subjective assessment of the difficulty of the exerted effort will be recorded after each task.

The physiotherapist will confirm that the patient is breathing rhythmically during the exercise, does not tense his/her whole body, and has complete muscle relaxation during the relaxation phase.

*Evaluation of the Myocyte Injury Markers.* Although the immunochemical determination of enzymatic isoforms specific to various tissues is out of the scope of classic diagnostic methods, it allows the origin of these isoforms to be identified, especially those derived from the skeletal muscles and the myocardium. Therefore, the following parameters will be determined in serum by means of ELISA:

(1) Creatine kinase (CK), namely, its muscular isozyme, CKMM [43]

(2) Lactate dehydrogenase 5 (LDH5) [44]

(3) Myoglobin

(4) Carbonic anhydrase III [45]

The muscular levels of carbonic anhydrase III and myoglobin will be expressed as the ratio of these two parameters as this characterizes skeletal muscles more accurately and makes it possible to distinguish between myoglobin of muscular origin and myoglobin of myocardial origin.

In the rehabilitation group, the therapeutic process will include cardiac rehabilitation according to the protocol used routinely at the Center for Heart Disease, 4th Military Hospital of Wrocław, while standard pharmacotherapy will be administered only in the control group.

The rehabilitation protocol, adjusted to clinical status, individual needs, and physical capabilities of the patient, consists of varying levels of exercise intensity. Once the stabilization of clinical status is achieved and absolute physical exercise contraindications are excluded, usually on the 2nd or 3rd day of hospitalization, cardiac rehabilitation will be prescribed by the physician in charge. The rehabilitation will be continued throughout the whole hospital stay.

The physical therapist will plan the individualized program of cardiac rehabilitation intended to minimize adverse outcomes of decompensation and physical deconditioning, helping the patient to achieve functional self-sufficiency.

Each rehabilitation session is conducted with supervision and monitoring of the following hemodynamic parameters: blood pressure (BP), heart rate (HR), oximetry, and frequency of breath.

The rehabilitation protocol comprises the following:

- Respiratory exercises
- Simple movement and activation of small muscles of lower and upper extremities alone and in combination
- Assisted exercises (with the help of a physiotherapist), if necessary
- Short-term isometric exercises
- Active dynamic exercises
- Flexibility exercises
- Strengthening exercises focused on the lower extremities using the patient's body weight as resistance [46]

The physical rehabilitation program will be conducted with very low intensity, short duration, and a properly adjusted recovery phase; it will be performed in a lying, sitting, or standing position.

The rehabilitation sessions will initially be conducted 2-3 times per day for 10 minutes at a time. As the patient's clinical condition and well-being improve, in the following days, the duration of rehabilitation will be extended to 20–25 minutes twice a day.

## 4. Discussion

The hemodynamic disorders observed in AHF are characterized by severe hemodynamic and neurohormonal abnormalities, including pulmonary and systemic congestion due to the elevated filling pressures, peripheral hypoperfusion due to decreased cardiac output, and vasoconstriction-related increase in systemic vascular resistance [4, 6]. Both venous congestion and peripheral hypoperfusion can lead to organ injury, dysfunction, and failure. They activate inflammatory, neurohormonal, and oxidative stress mechanisms, which cause endothelial dysfunction, ischemia, fibrosis, and cell death due to apoptosis or necrosis in multiple tissues and organs [6, 20]. Inadequate tissue perfusion itself results in local hypoxia and impaired aerobic metabolism, leading to decreased energy production and finally cell injury and death [4, 47].

Therefore, the episode of AHF can bring about multiple organ failures and involve skeletal muscles as well. This is of vital importance because respiratory muscles may become affected as well; their involvement enhances the preexisting imbalance between the perfusion of muscle tissue and the oxygen demand of myocytes, resulting from hypoxia and reflex stimulation of the respiratory drive.

(1) The primary aim of this study is to comprehensively evaluate the skeletal muscles (at a functional and tissue level) during ADHF.

By examining the myocyte injury markers, muscle perfusion, and oxygenation, the aim is to determine whether ADHF causes damage to the skeletal muscles.

Although biomarkers used in everyday practice reflect a degree of injury in various organs (e.g., kidneys and liver) [8, 30], most of them are not specific and thus their presence (e.g., creatine kinase, lactate dehydrogenase, and myoglobin) can correspond to the injury of skeletal muscles as well [43–45]. This is even more likely, as skeletal muscles constitute a considerable percentage of the total mass of the human body; therefore, even a slight muscular injury can modulate the concentrations of these biomarkers. Consequently, we are going to determine the levels of biomarkers reflecting the degree of cell necrosis and failure of various organs in order to assess the severity of skeletal muscle injury.

Taking into account the skeletal myopathy in patients with stable CHF [21, 22], it can be assumed that immobilization in the acute phase of the disease and limited mobility during hospitalization result in a continued decline in muscle dysfunction.

As a consequence, the cascade of these adverse ADHD factors can drastically increase the risk of functional impairment or even disability.

This is why we want to observe whether muscles constitute part of the multiple organ dysfunction during ADHF.

The majority of patients with HF are elderly and have multiple comorbidities [48]. Frailty is common, being seen in more than 50% of ADHF patients [49].

Frailty and comorbidities that exist together contribute to disabilities, frequent rehospitalizations, and poor prognosis [50, 51].

Additionally, we want to evaluate whether there is a relationship between CO and SVR and the parameters determining skeletal muscles.

(2) The second aim of this project is to assess the changes in these parameters obtained in three studies, at admission, at discharge, and 30 days after the discharge.

Skeletal muscles play an important role in many HF-associated pathologies, as their normal function determines the physical fitness and capability of the patients and underlies the feeling of independence in everyday life [21].

Earlier studies showed that a patient's decreased physical functioning is linked with a greater risk of rehospitalization [52, 53].

Moreover, a considerable decrease in the functional performance can be long-lasting, while recovery to functional self-sufficiency may be difficult to achieve or even impossible [54]. Therefore, in addition, we would like to evaluate the elements of functional capacity (mobility, strength, and endurance) at discharge and during recovery and clinical stabilization, which accounts for a period of 30 days after discharge.

Evaluation of the skeletal muscles will be performed on the background of clinical status in the acute phase, at discharge and 30 days after discharge.

(3) Another aim of the study is to assess the influence of 3 different types of physical exercises on muscle tissue perfusion, muscle fatigue, and vital/hemodynamic parameters (SV, CO, SVR, HR, BP, respiratory rate, and saturation).

We have selected exercises based on simple movements, which should be sufficiently safe to be applied in the first physiotherapeutic procedures/tasks at the beginning of rehabilitation. The purpose of these exercises is to prevent the negative effects of immobilization and the potential adverse effects of hypoperfusion and hypovolemia on the skeletal muscles.

Assisted and dynamic exercises as well as quadriceps muscle isometric contractions that are short in duration are proposed during the early rehabilitation of ADHF patients [55]. Moreover, they provide the basis for further modifications, extensions, and intensification of the rehabilitation program.

Because of the specificity of the disease, it is not possible to use a typical protocol to evaluate muscle fatigue that involves constantly maintained isometric tension in ADHF patients. In this test, the electromyographic (EMG) activity of the quadriceps muscle is registered during the isometric knee extension at 60% of the MVC until exhaustion is reached [56].

The dynamic exercise was also planned (the selection of patient position and muscle group exercised, range of motion in a lying position with bed headboard raised, and dynamic knee extension in the knee joint in an incomplete range of movement) in order to minimize the effort that the patient must exert to do the exercise on the one hand and to stimulate muscle flow/activate the patient on the other hand.

The next goal of our research is to assess the safety and impact of early in-hospital rehabilitation on hemodynamic parameters and the functional capacity of patients hospitalized due to AHF. Nowadays, increasing attention is being paid to the negative effects of hospitalization in elderly patients with chronic diseases. One of these is the low level of patient mobility and level of bed rest required (20% of patients do not move out of bed and 30% only walk or move about their room). In addition to the consequences associated with the acute phase of the disease and psychological stress, the in-hospital negative functional decline affects the health and psychophysical well-being of patients in early convalescence, leading to the so-called posthospital syndrome [57, 58].

Physical impairments that accompany CHF significantly worsen with decompensation. Furthermore, they are increased due to the prolonged immobility associated with the hospital environment, subsequently resulting in significant impairments in physical function. Acute immobility results in a decrease in existing functional capacity [29].

Therefore, the rehabilitation program should be implemented as soon as possible after achieving clinical stabilization [26]. Scientific reports on exercise interventions during acute decompensation in heart failure are very limited. The first publications appeared a few years ago, which demonstrated the safety and efficacy of cardiac rehabilitation in patients hospitalized due to AHF [58, 59].

The subsequent projects presented multidomain programs for early posthospital rehabilitation after AHF and its benefits. The important role of rehabilitation in the care of elderly patients with frailties and comorbidities during and after the AHF episode was emphasized.

In the Polish recommendations, a model for implementing comprehensive cardiac rehabilitation of patients with AHF was created and proposed. The rehabilitation program proposed here is in line with the above-mentioned recommendations [46]. Its purpose is the safe, supervised, and monitored mobilization/rehabilitation of the patient, minimizing the negative effects of HF decompensation and hospitalization, and maintaining or restoring the functional self-sufficiency of the patient [23–25].

Comprehensive interdisciplinary studies of the peripheral pathomechanisms activated in the skeletal muscles of patients with AHF seem crucial, at least in the context of developing targeted therapeutic strategies.

## Figures and Tables

**Table 1:** Protocol of the study.

Methods of evaluation	1st day of hospitalization	1st-2nd days of hospitalization	Last day of hospitalization, discharge from hospital	30 days after discharge
	Examination	Examination	Examination	Examination
Clinical examination	**+**		**+**	**+**
Hemodynamic assessment		**+**	**+**	**+**
Examination of muscle function		**+**	**+**	**+**
Examination of tissue perfusion		**+**	**+**	**+**
Markers of muscular injury	**+**		**+**	**+**

## References

[1] Ponikowski P., Voors A. A., Anker S. D. (2016). 2016 ESC Guidelines for the diagnosis and treatment of acute and chronic heart failure: the Task Force for the diagnosis and treatment of acute and chronic heart failure of the European Society of Cardiology (ESC) developed with the special contribution of the Heart Failure Association (HFA) of the ESC. *European Heart Journal*.

[2] Rudiger A., Harjola V.-P., Müller A. (2005). Acute heart failure: Clinical presentation, one-year mortality and prognostic factors. *European Journal of Heart Failure*.

[3] Ambrosy A. P., Fonarow G. C., Butler J. (2014). The global health and economic burden of hospitalizations for heart failure. *Journal of the American College of Cardiology*.

[4] Harjola V.-P., Mullens W., Banaszewski M. (2017). Organ dysfunction, injury and failure in acute heart failure: from pathophysiology to diagnosis and management. A review on behalf of the Acute Heart Failure Committee of the Heart Failure Association (HFA) of the European Society of Cardiology (ESC). *European Journal of Heart Failure*.

[5] Lauten A., Ferrari M., Goebel B. (2011). Microvascular tissue perfusion is impaired in acutely decompensated heart failure and improves following standard treatment. *European Journal of Heart Failure*.

[6] Gheorghiade M., Pang P. S. (2009). Acute Heart Failure Syndromes. *Journal of the American College of Cardiology*.

[7] Legrand M., Mebazaa A., Ronco C., Januzzi J. L. (2014). When cardiac failure, kidney dysfunction, and kidney injury intersect in acute conditions: The case of cardiorenal syndrome. *Critical Care Medicine*.

[8] Bart B. A., Goldsmith S. R., Lee K. L. (2012). Cardiorenal rescue study in acute decompensated heart failure: Rationale and design of CARRESS-HF, for the heart failure clinical research network. *Journal of Cardiac Failure*.

[9] Coats A. J. S., Clark A. L., Piepoli M., Volterrani M., Poole-Wilson P. A. (1994). Symptoms and quality of life in heart failure: the muscle hypothesis. *British Heart Journal*.

[10] Coats A. J. S. (1996). The ‘Muscle Hypothesis’ of chronic heart failure. *Journal of Molecular and Cellular Cardiology*.

[11] Piepoli M. F., Kaczmarek A., Francis D. P. (2006). Reduced peripheral skeletal muscle mass and abnormal reflex physiology in chronic heart failure. *Circulation*.

[12] von Haehling S., Lainscak M., Springer J., Anker S. D. (2009). Cardiac cachexia: a systematic overview. *Pharmacology & Therapeutics*.

[13] Kinugawa S., Takada S., Matsushima S., Okita K., Tsutsui H. (2015). Skeletal muscle abnormalities in heart failure. *International Heart Journal*.

[14] Sullivan M. J., Green H. J., Cobb F. R. (1990). Skeletal muscle biochemistry and histology in ambulatory patients with long-term heart failure. *Circulation*.

[15] Sabbah H. N., Hansen-Smith F., Sharov V. G. (1993). Decreased proportion of type I myofibers in skeletal muscle of dogs with chronic heart failure. *Circulation*.

[16] Drexler H., Riede U., Münzel T., König H., Funke E., Just H. (1992). Alterations of skeletal muscle in chronic heart failure. *Circulation*.

[17] Ponikowski P. P., Chua T. P., Francis D. P., Capucci A., Coats A. J. S., Piepoli M. F. (2001). Muscle ergoreceptor overactivity reflects deterioration in clinical status and cardiorespiratory reflex control in chronic heart failure. *Circulation*.

[18] Afilalo J., Karunananthan S., Eisenberg M. J., Alexander K. P., Bergman H. (2009). Role of frailty in patients with cardiovascular disease. *American Journal of Cardiology*.

[19] Newman A. B., Gottdiener J. S., McBurnie M. A. (2001). Associations of Subclinical Cardiovascular Disease With Frailty. *The Journals of Gerontology. Series A, Biological Sciences and Medical Sciences*.

[20] Casado Cerrada J., Zabaleta Camino J. P., Fontecha Ortega M. (2016). Target organ damage in acute heart failure. *Revista Clínica Española*.

[21] Middlekauff H. R. (2010). Making the case for skeletal myopathy as the major limitation of exercise capacity in heart failure. *Circulation: Heart Failure*.

[22] Okita K., Kinugawa S., Tsutsui H. (2013). Exercise intolerance in chronic heart failure - Skeletal muscle dysfunction and potential therapies. *Circulation Journal*.

[23] Reeves G. R., Whellan D. J., Duncan P. (2017). Rehabilitation Therapy in Older Acute Heart Failure Patients (REHAB-HF) trial: Design and rationale. *American Heart Journal*.

[24] Acanfora D., Scicchitano P., Casucci G. (2016). Exercise training effects on elderly and middle-age patients with chronic heart failure after acute decompensation: A randomized, controlled trial. *International Journal of Cardiology*.

[25] Reeves G. R., Whellan D. J., O'Connor C. M. (2017). A novel rehabilitation intervention for older patients with acute decompensated heart failure: the REHAB-HF pilot study. *JACC: Heart Failure*.

[26] Piepoli M. F., Conraads V., CorrÁ U. (2011). Exercise training in heart failure: From theory to practice. A consensus document of the heart failure association and the european association for cardiovascular prevention and rehabilitation. *European Journal of Heart Failure*.

[27] Piepoli M. F. (2013). Exercise training in chronic heart failure: mechanisms and therapies. *Netherlands Heart Journal*.

[28] Conraads V. M., Deaton C., Piotrowicz E. (2012). Adherence of heart failure patients to exercise: barriers and possible solutions: a position statement of the Study group on exercise training in heart failure of the heart failure association of the european society of cardiology. *European Journal of Heart Failure*.

[29] Fleg J. L., Cooper L. S., Borlaug B. A. (2015). Exercise training as therapy for heart failure current status and future directions. *Circulation: Heart Failure*.

[30] Heil B., Tang W. H. W. (2015). Biomarkers: Their potential in the diagnosis and treatment of heart failure. *Cleveland Clinic Journal of Medicine*.

[31] Myers J. N., Gujja P., Neelagaru S., Hsu L., Burkhoff D. (2011). Noninvasive measurement of cardiac performance in recovery from exercise in heart failure patients. *Clinics*.

[32] Gielerak G., Krzesiński P., Piotrowicz E., Piotrowicz R. (2013). The usefulness of impedance cardiography for predicting beneficial effects of cardiac rehabilitation in patients with heart failure. *BioMed Research International*.

[33] Aizawa Y., Takatsuki S., Kashimura S. (2014). Thoracic impedance as a therapeutic marker of acute decompensated heart failure. *International Journal of Cardiology*.

[34] Sadauskas S., Naudžiūnas A., Unikauskas A., Mašanauskienė E., Bakšytė G., Macas A. (2016). Applicability of impedance cardiography during heart failure flare-ups. *Medical Science Monitor*.

[35] Farina D. (2006). Interpretation of the surface electromyogram in dynamic contractions. *Exercise and Sport Sciences Reviews*.

[36] González-Izal M., Malanda A., Navarro-Amézqueta I. (2010). EMG spectral indices and muscle power fatigue during dynamic contractions. *Journal of Electromyography & Kinesiology*.

[37] Mau-Moeller A., Jacksteit R., Jackszis M. (2017). Neuromuscular function of the quadriceps muscle during isometric maximal, submaximal and submaximal fatiguing voluntary contractions in knee osteoarthrosis patients. *PLoS ONE*.

[38] Rikli R. E., Jones C. J. (1999). Development and validation of a functional fitness test for community-residing older adults. *Journal of Aging and Physical Activity*.

[39] Wegrzynowska-Teodorczyk K., Mozdzanowska D., Josiak K. (2016). Could the two-minute step test be an alternative to the six-minute walk test for patients with systolic heart failure?. *European Journal of Preventive Cardiology*.

[40] Ding H., Wang G., Lei W. (2001). Non-invasive quantitative assessment of oxidative metabolism in quadriceps muscles by near infrared spectroscopy. *British Journal of Sports Medicine*.

[41] Tew G. A., Ruddock A. D., Saxton J. M. (2010). Skin blood flow differentially affects near-infrared spectroscopy-derived measures of muscle oxygen saturation and blood volume at rest and during dynamic leg exercise. *European Journal of Applied Physiology*.

[42] De Blasi R., Luciani R., Punzo G. (2009). Microcirculatory changes and skeletal muscle oxygenation measured at rest by non-infrared spectroscopy in patients with and without diabetes undergoing haemodialysis. *Critical Care*.

[43] Naclerio F., Larumbe-Zabala E., Cooper R., Allgrove J., Earnest C. P. (2015). A multi-ingredient containing carbohydrate, proteins L-glutamine and L-carnitine attenuates fatigue perception with no effect on performance, muscle damage or immunity in soccer players. *PLoS ONE*.

[44] Vaidya H. C., Dietzler D. N., Ladenson J. H. (1986). Quantitation of serum lactate dehydrogenase-5 with monoclonal antibodies. *Clinica Chimica Acta*.

[45] Staunton L., Zweyer M., Swandulla D., Ohlendieck K. (2012). Mass spectrometry-based proteomic analysis of middle-aged vs. aged vastus lateralis reveals increased levels of carbonic anhydrase isoform 3 in senescent human skeletal muscle. *International Journal of Molecular Medicine*.

[46] Piotrowicz R., Jegier A., Szalewska D.

[47] Hogan C. J., Ward K. R., Kontos M. C., Thacker L. R., Pittman R. (2012). Peripheral tissue oxygenation improves during ED treatment of acute heart failure. *The American Journal of Emergency Medicine*.

[48] Ahluwalia S. C., Gross C. P., Chaudhry S. I., Leo-Summers L., Van Ness P. H., Fried T. R. (2011). Change in comorbidity prevalence with advancing age among persons with heart failure. *Journal of General Internal Medicine*.

[49] Reeves G. R., Whellan D. J., Patel M. J. (2016). Comparison of frequency of frailty and severely impaired physical function in patients ≥60 years hospitalized with acute decompensated heart failure versus chronic stable heart Failure with Reduced and Preserved Left Ventricular Ejection Fraction. *American Journal of Cardiology*.

[50] Murad K., Kitzman D. W. (2012). Frailty and multiple comorbidities in the elderly patient with heart failure: Implications for management. *Heart Failure Reviews*.

[51] Vidán M. T., Blaya-Novakova V., Sánchez E., Ortiz J., Serra-Rexach J. A., Bueno H. (2016). Prevalence and prognostic impact of frailty and its components in non-dependent elderly patients with heart failure. *European Journal of Heart Failure*.

[52] Kommuri N. V. A., Johnson M. L., Koelling T. M. (2010). Six-minute Walk Distance Predicts 30-Day Readmission in Hospitalized Heart Failure Patients. *Archives of Medical Research*.

[53] Delgado Parada E., Suárez García F. M., López Gaona V., Gutiérrez Vara S., Solano Jaurrieta J. J. (2012). Mortality and functional evolution at one year after hospital admission due to heart failure (HF) in elderly patients. *Archives of Gerontology and Geriatrics*.

[54] Bodilsen A. C., Pedersen M. M., Petersen J. (2013). Acute hospitalization of the older patient: Changes in muscle strength and functional performance during hospitalization and 30 days after discharge. *American Journal of Physical Medicine & Rehabilitation*.

[55] Komi P. V., Viitasalo J. H. T. (1976). Signal Characteristics of EMG at Different Levels of Muscle Tension. *Acta Physiologica Scandinavica*.

[56] Zisberg A., Syn-Hershko A. (2016). Factors related to the mobility of hospitalized older adults: A prospective cohort study. *Geriatric Nursing*.

[57] Zisberg A., Shadmi E., Sinoff G., Gur-Yaish N., Srulovici E., Admi H. (2011). Low mobility during hospitalization and functional decline in older adults. *Journal of the American Geriatrics Society*.

[58] Scrutinio D., Passantino A., Catanzaro R. (2012). Inpatient cardiac rehabilitation soon after hospitalization for acute decompensated heart failure: A propensity score study. *Journal of Cardiopulmonary Rehabilitation and Prevention*.

[59] Babu A., George M., Guddattu V., Maiya A., Padmakumar R. (2011). Effects of combined early in-patient cardiac rehabilitation and structured home-based program on function among patients with congestive heart failure: a randomized ontrolled trial. *Heart Views*.

